# Modulation of choroidal neovascularization by subretinal injection of retinal pigment epithelium and polystyrene microbeads

**Published:** 2009-01-21

**Authors:** Ingo Schmack, Lennart Berglin, Xiaoyan Nie, Jing Wen, Shin J. Kang, Adam I Marcus, Hua Yang, Michael J. Lynn, Judith A Kapp, Hans E. Grossniklaus

**Affiliations:** 1 Department of Ophthalmology, Emory University School of Medicine, Atlanta, GA; 2 Department of Ophthalmology, Ruprecht-Karls-University, Heidelberg, Germany; 3 Department of Ophthalmology, St. Erik’s Eye Hospital, Karolinska Institutet, Stockholm, Sweden; 4 Winship Cancer Institute, Emory University School of Medicine, Atlanta, GA; 5 Department of Biostatistics, Emory University School of Medicine, Atlanta, GA; 6 Department of Ophthalmology, University of Alabama School of Medicine, Birmingham, AL

## Abstract

**Purpose:**

The study was conducted to create a rapidly developing and reproducible animal model of subretinal choroidal neovascularization (CNV) that allows a time-dependent evaluation of growth dynamics, histopathologic features, and cytokine expression.

**Methods:**

C57BL/6 and chemoattractant leukocyte protein-2 deficient (∆Ccl-2) mice were studied. Mice received single or combined subretinal injections of cultured retinal pigment epithelium (RPE; C57BL/6-derived), polystyrene microbeads, or phosphate buffer solution (PBS). Fluorescence angiograms were performed over a period of 3 weeks. Mice were euthanized on post inoculation day 3, 7, 10, 14,  or 21, and their eyes were evaluated by light, confocal, and electron microscopy.

**Results:**

CNV membranes occurred in all study groups with an overall incidence of 94.3%. They extended in the subretinal space through central breaks in Bruch’s membrane. CNV lesions were characterized by dynamic changes such as initiation, active inflammatory, and involution stages. CNV thickness peaked around PI day 7 and was greater in mice that received combined injections of RPE and microbeads or RPE cells alone. Small lesions developed in the control groups (microbeads or PBS only), in ∆Ccl-2, and old C57BL/6 mice. Variable expression of cytokines and growth factors was detected within the membranes.

**Conclusions:**

Our murine model represents a reliable approach inducing CNV growth by subretinal injection of either RPE cells alone or RPE cells and microbeads. The development of CNV lesions is a dynamic process that relies in part on macrophage trafficking and age.

## Introduction

Choroidal neovascularization (CNV) is a nonspecific, stereotypical, ocular wound-healing response to damage of the retinal pigment epithelium (RPE)-Bruch’s membrane-choroidal complex [1,2]. It has been observed in patients with age-related macular degeneration (AMD), pathologic myopia, pseudoxanthoma elasticum (Grönblad-Strandberg-Syndrome), presumed ocular histoplasmosis (POHS), and other subtypes of posterior uveitis, as well as in patients who have had ocular trauma [1–5]. CNV lesions usually arise from the choriocapillaris with subsequent extension through breaks of Bruch’s membrane in the subRPE and subretinal space [1,2,5–8]. In general, two main CNV growth patterns can been distinguished: type 1 growth pattern (sub-RPE), which involves CNV extension in the plane between Bruch’s membrane and the RPE; and type 2 growth pattern (subretinal), which is CNV invasion into the subretinal space between the RPE and the photoreceptor outer segments [1,2,9,10].

CNV evolution is a dynamic process comprised of initiation, active inflammatory, and inflammatory inactive (involutional) phases, comparable to dermal wound healing [11–13]. It is regulated by a close interaction of various cell types, cytokines, growth factors, and components of the innate immune system resulting in a fibrovascular granulation tissue [8,11,12,14–19]. Morphologic and ultrastructural studies have shown that the cellular and extracellular components of CNV lesions (i.e., RPE, macrophages, fibrocytes, and vascular endothelium, fibrin) are quite similar, regardless of the underlying disease [1,2,10,20].

However, histopathologic and immunohistochemical investigations performed on post-mortem eyes with AMD and surgically excised CNV specimens have limitations. Post-mortem eyes usually display late stages of CNV and do not provide reliable information of early changes occurring in CNV evolution (initiation and active inflammatory stages) because of a considerable time-gap between onset of the disease and harvest of the tissue [20,21]. Therefore, animal models of experimental CNV are needed to better investigate the pathobiology of the lesion in early, mid, and late stages during CNV formation. Current CNV models are based on mechanical and transgenic approaches such as enzymatic or traumatic disruption of the RPE and Bruch’s membrane (i.e., laser photocoagulation) as well as vector-driven (e.g., matrigel, microspheres, adeno-associated virus) delivery of pro- and anti-angiogenic cytokines into the vitreous, subretinal, or suprachoroidal space [22–34]. These models usually result in small-sized CNV lesions (especially after laser injury) that primarily extend in the subRPE space (type I growth pattern) only.

The purpose of this study was to introduce a rapidly developing and highly reproducible animal (mouse) CNV model, which is characterized by growth dynamics, histopathologic, and ultrastructural features similar to subretinal (type II growth pattern) CNV lesions. It is based on a traumatic injury to Bruch’s membrane secondary to subretinal injections of murine RPE cells and polystyrene microbeads. RPE cells were used since they are multifunctional cells involved in CNV formation by secretion of various cytokines and growth factors. Microbeads on the other hand represent chemically inert particles that can potentially serve as vehicles for delivery of cytokines or anti-angiogenic agents to the induced membranes. Overall, we investigated the influence of RPE cells, alone or in combination with microbeads, as well as the impact of impaired macrophage recruitment and aging on CNV formation.

## Methods

### Experimental animals

The study was conducted after approval of the Institutional Animal Care and Use Committee of the Emory University. All animals were treated in accordance with the principles described in The Guiding Principles in the Care and Use of Animals (NIH), and the ARVO Statement for the Use of Animals in Ophthalmic and Vision Research. Female C57BL/6 (stock #000664) and chemoattractant leukocyte protein-2 deficient (∆Ccl-2; stock #004434) mice were purchased from The Jackson Laboratories (Bar Harbor, ME). They were either 2-month-old C57BL/6 and ∆Ccl-2 mice (young), weighing 18–20 g, or 12-month-old C57BL/6 mice (old), weighing 25–30 g. Weights given are what the mice weighed at the onset of the study. ∆Ccl-2 mice were used since they demonstrate an impaired recruitment of monocytes and macrophages despite normal numbers of circulating leukocytes and resident macrophages compared to wild-type mice (i.e., C57BL/6) [35]. All mice were housed in an institutional animal care facility in routine polycarbonate cages with free access to food (regular rodent diet) and water, and maintained with a 12 h:12 h light-dark cycle.

### Experimental design

Mice were divided in 4 study groups with regard to the subretinal injection technique (RPE cells only, RPE cells combined with microbeads), genetic background (C57BL/6, ∆Ccl-2), and age (C57BL/6; 2-month-old, classified as young, or 12-month-old, categorized as old). Controls were 2-month old C57BL/6 mice who received subretinal injections of phosphate buffer solution (PBS; KH_2_PO_4_ [0.144 g/l], NaCl [9.0 g/l], and Na_2_HPO_4_ [0.795 g/l]) or polystyrene microbeads (Table 1). Each group was composed of 25 animals. Five mice per group were sequentially euthanized at post-inoculation (PI) day 3, 7, 10, 14, and 21. Both eyes were enucleated and evaluated for CNV growth by light microscopy (right eye; n=5/group), confocal scanning laser microscopy (left eye; n=2/group), and transmission and scanning electron microscopy (left eye; n=3/group). Fluorescein angiograms were performed in single animals (n=2/group) at PI day 3, 7, 10, 14, and 21.

**Table 1:** Characteristics of the study and control groups

**Mice**			**Injected material**		
**Group**	**Genotype**	**Age (month)**	**Agent**	**Volume (µl)**	**Concentration**
Study group 1	C57BL/6	2	RPE	50	0.5×10^5^/µl
Study group 2	C57BL/6	2	RPE+MB	50	0.5×10^5^/µl
Study group 3	Ccl-2^−/−^	2	RPE+MB	50	0.5×10^5^/µl
Study group 4	C57BL/6	12	RPE+MB	50	0.5×10^5^/µl
Control group 1	C57BL/6	2	PBS	50	-
Control group 2	C57BL/6	2	MB	50	0.5×10^5^/µl

The table summarizes the characteristics of the four study and the two control groups and provides information on the subretinal injected agents. The animal groups were composed of 2-month-old C57BL/6 mice (study and control group 1+2), 12-month-old C57BL/6 mice (study group 4), and 2-month-old Ccl-2 knockout mice (Ccl-2^−/−^, study group 3). Choroidal neovascularization membranes were induced by subretinal injection of 50 µl of retinal pigment epithelium (RPE) cells alone or RPE cells and microbeads (MB) with a concentration of 0.5x10^5^/µl each. The control mice received subretinal injections (50 µl) of phosphate buffer solution (PBS) or microbeads (0.5x10^5^/µl) Abbreviations: Ccl-2 knockout mice (Ccl-2^−/−^); RPE represents retinal pigment epithelium (RPE); microbeads (MB); phosphate buffered solution (PBS); standard deviation (SD).

### Microbead preparation

Uniform (4.5 µm) polystyrene superparamagnetic microbeads (Dynabeads® M-450 Tosyl activated; Dynal Biotech LLC., Brown Deer, WI) were prepared as previously described [36]. In detail, microbeads were washed in 0.1 M phosphate buffer, pH 7.4 (2.62 g NaH_2_PO_4_xH_2_O [MW137.99], 14.42 g NaH_2_PO_4_x2 H_2_O8 [MW177.00]), resuspended in phosphate buffered saline (PBS) containing 0.1% (w/v) BSA (BSA Fraction V; Sigma-Aldrich Corp., St. Louis, MO), and incubated for 18 h at 37 °C to block the activated tyosyl groups. The microbeads were then washed twice in PBS containing 0.1% (w/v) BSA, once in 0.2 M Tris containing 0.1% (w/v) BSA, and stored in PBS containing 0.1% (w/v) BSA. Prior to injection, the microbeads were homogenized and a desired volume of microbeads was transferred to a 1.5 ml test tube and placed in a magnetic rack (Dynal MPC-Magnetic Particle Concentrator; Dynal Biotech LLC.) for 1 min. The supernatant was carefully discarded and the remaining microbeads were resuspended in 1,000 µl PBS containing 0.1% (w/v) BSA. Each step was repeated 5 times. Finally, the microbeads were counted and diluted with Hank’s Balanced Salt Solution (HBSS, 1X; Invitrogen Corp., Carlsbad, CA) to a working concentration of 0.5x10^5^ microbeads/µl.

### Isolation of murine RPE

**Figure 1:**
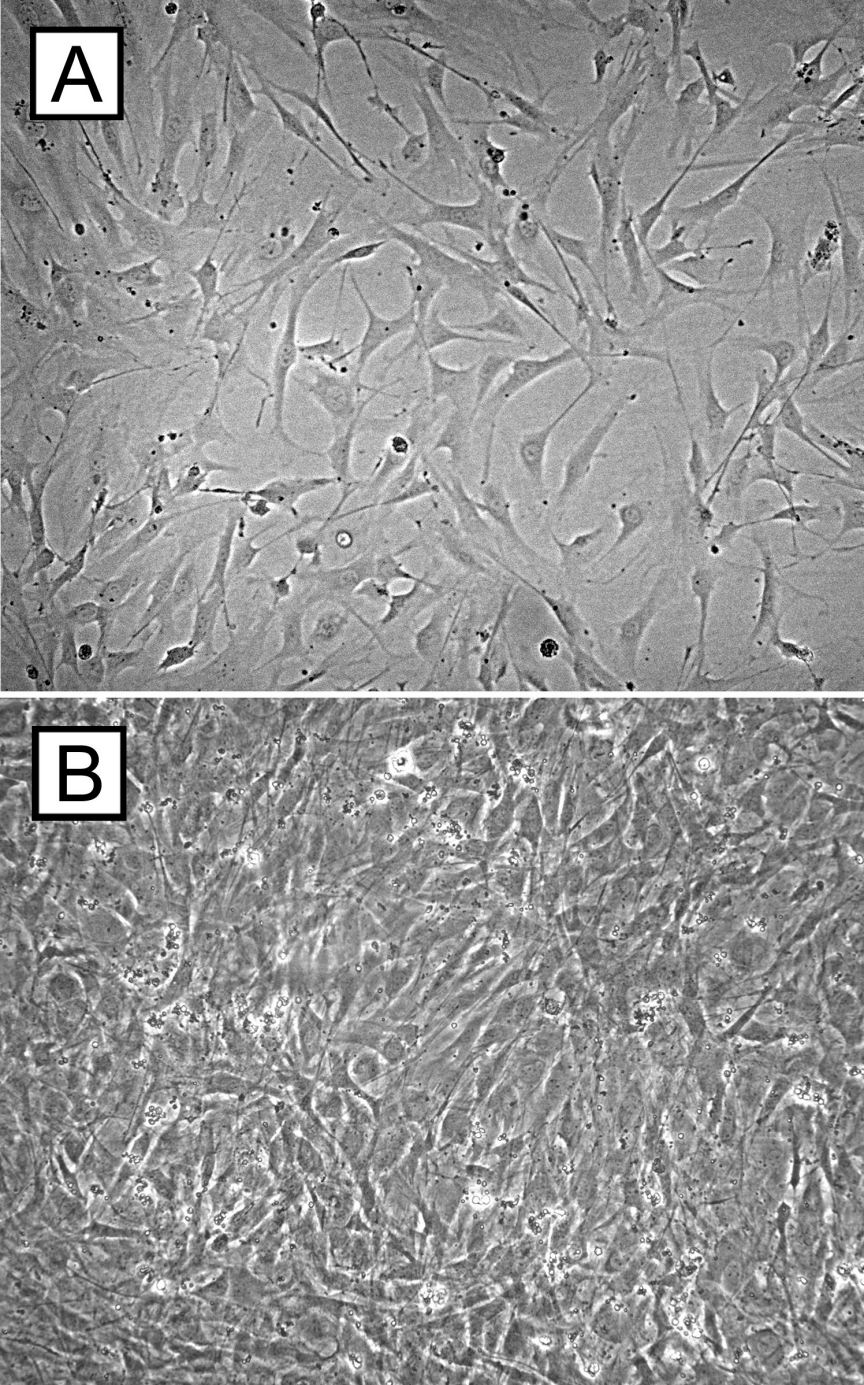
Cell culture experiments of retinal pigment epithelium cells. Retinal pigment epithelium (RPE) cells of C57BL/6 mice were seeded on tissue culture flasks containing RPE cell culture medium and cultured for up to 7 days till they were grown to confluence. The micrographs demonstrate C57BL/6 RPE cells after 3 (**A**) and 7 (**B**) days of cell culture.

Murine RPE cells were harvested from C57BL/6 mice. The freshly enucleated eyes (n=10) were submerged in 5% Betadine solution® (The Purdue Frederick Company, Stamford, CT) for 2–3 min, subsequently rinsed in sterile HBSS (2-times), and incubated in an enzymatic solution (pH 8.0) containing HBSS, 19.5 U/ml collagenase, 38 U/ml hyaluronidase, and 0.1% trypsin, at 37 °C for 40 min. Following enzymatic digestion, sclera and choroid were dissected from the remaining eye by a circular incision about 1 mm posterior to the limbus. The RPE monolayer was gently scraped from the retina, which remained attached to the vitreous. The RPE cell suspension was placed in a 50 ml tube filled with Dulbecco’s Modified Eagle Medium/F-12 RPE cell culture medium (1X; Gibco, Grand Island, NY), 100 IU/µl penicillin sodium, 100 µg/ml  streptomycin sulfate, 25 µg/ml amphotericin B, and 5% (vol/vol) fetal bovine serum (Sigma-Aldrich Corp., St. Louis, MO). The RPE cells were centrifuged, resuspended in fresh RPE culture medium, counted, seeded on 25 cm^2^ tissue culture flasks, and incubated at 37 °C in a humidified 5% CO_2_ atmosphere till grown to confluence (mean 4 to 6 days). Approximately 10^5^ RPE cells/eye were routinely obtained. The cells in the flasks were checked daily for any signs of contamination, and the RPE growth medium was changed every other day. Once the flask was covered with an RPE cell monolayer (Figure 1), cells were trypsinized with 0.05% Trypsin EDTA (1X; Gibco) for 10 to 15 min, centrifuged for 5 min at 200x g, washed 3 times in HBSS, and counted after staining with trypan blue (staining of dead cells). Subsequently, the RPE cell pellet was resuspended in HBSS, diluted to a final working concentration of 0.5x10^5^ cells/µl, and stored on ice. Prior to injection a desired volume of the RPE cell suspension was mixed with microbeads at a ratio of 1:1 (0.5x10^5^ cells/μl with 0.5x10^5^ microbeads/μl).

### Subretinal injection technique

**Figure 2:**
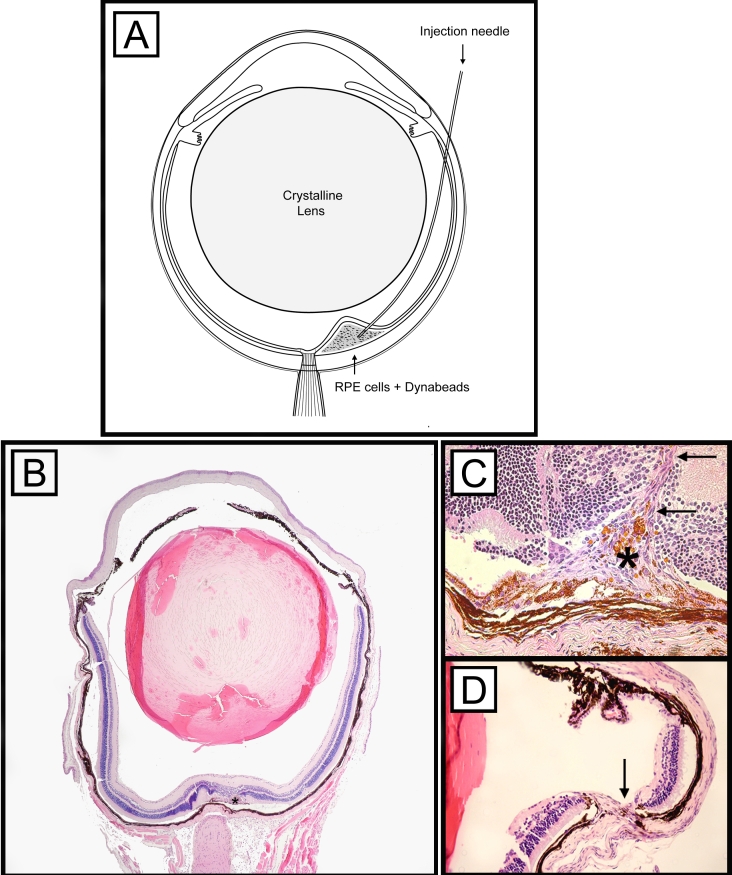
Illustration of the subretinal injection technique. A bent needle was inserted through a sclerotomy at 10 o’clock next to the limbus and pushed forward in a tangential direction toward the retina without touching the crystalline lens (**A**). Three to 21 days after inoculation, choroidal neovascularization membranes (asterisk) could be identified in the subretinal space next to the optic nerve by light microscopy (**B, C**). The retinal (**C**) and sceral (**D**) insertion sites are highlighted by arrows. **B-D:** Periodic acid-Schiff (PAS) staining was used and original magnifications are 4X (**B**) and 20X (**C,D**).

**Figure 3:**
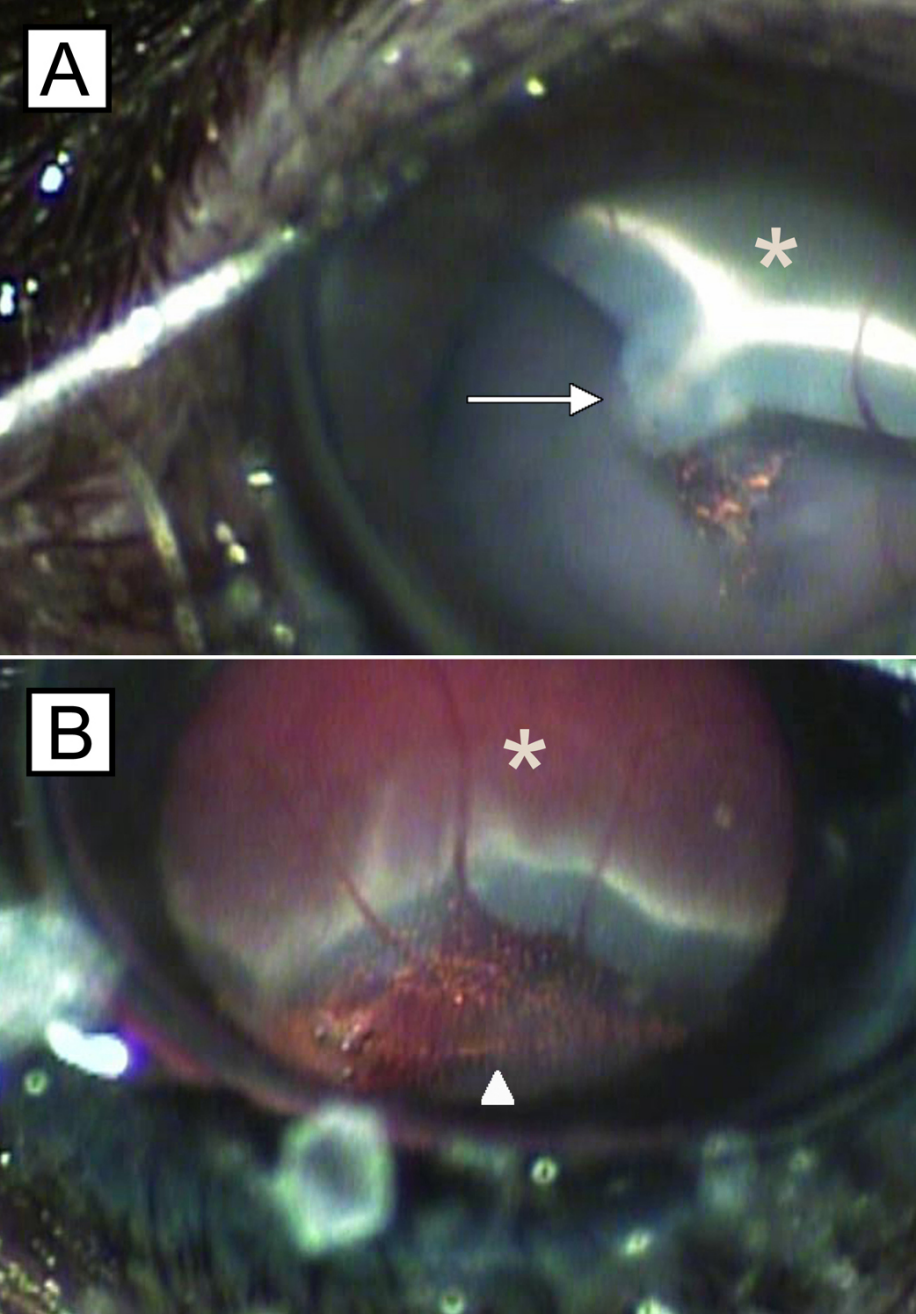
Mouse eyes showing retinal detachment after subretinal injection. Fundus photographs demonstrate circumscribed areas of retinal detachment (asterisks) following subretinal injection of retinal pigment epithelium (RPE) cells and microbeads (**A,B**). Occasionally, microbeads (**B**, arrowhead) and RPE cells could be observed in the vitreous cavity next to the injection site (**A**, arrow).

Injections were performed in a similar fashion as previously described [36]. Briefly, animals were anesthetized with an intramuscular administration of a mixture of 90 mg/kg ketamine hydrochloride (Sigma-Aldrich, St. Louis, MO) and 10 mg/kg xylazine hydrochloride (Sigma-Aldrich). Pupils were dilated with an application of 0.5% tropicamide, (Tropicacyl^®^; Akorn, Inc., Buffalo Grove, IL) and 0.5% proparacaine hydrochloride (Proparacain^®^; Akorn, Inc.) was applied to each eye. The eyes were gently proptosed, and a 30 gauge needle was used to perform a shelving puncture of the sclera at 10 o’clock 1.5 mm posterior to the limbus. A bent 33 gauge blunt needle attached to a 10 μl Hamilton syringe was passed through the sclerotomy in a tangential direction toward the posterior pole without touching the crystalline lens and placed on the inner surface of the retina next to the optic disc (Figure 2A). The retina was perforated and 2 µl PBS or RPE cells or RPE cells and microbeads was injected into the subretinal space (Figure 2B). The success of each injection was confirmed by gently pressing a microscope coverslip on the cornea, and the fundus was evaluated, using a surgical microscope (Figure 3), for signs of retinal detachment or subretinal hemorrhages . Excluded from the study were eyes that demonstrated severe subretinal or intravitreal hemorrhages (complete hemorrhage of the subretinal bleb or vitreous cavity) and mice that did not show a retinal detachment. Topical gentamicin sulfate 0.03% (Akorn Inc.) were applied to eyes at the end of each procedure.

### Fluorescein angiography

After subretinal injection, fluorescein angiograms were obtained on days 3, 7, 10, 14, and 21. Briefly, mice (n=2/group) were anesthetized with ketamine and xylazine, and the pupils were dilated with 0.5% tropicamide eye drops. Next, 0.1 ml sodium fluorescein (25%, Akorn Inc.) was injected intraperitoneally, and serial angiograms were captured at approximately 3 to 10 min after dye injection using a Nikon D1 (Nikon, Melville, NY) digital camera (16X magnifiers, power supply flash 3) attached to a Zeiss photo-slit-lamp. Vascular leakage was defined as the presence of a hyperfluorescent spot that increased in size over time.

### Light microscopy and quantitative CNV thickness measurements

**Figure 4:**
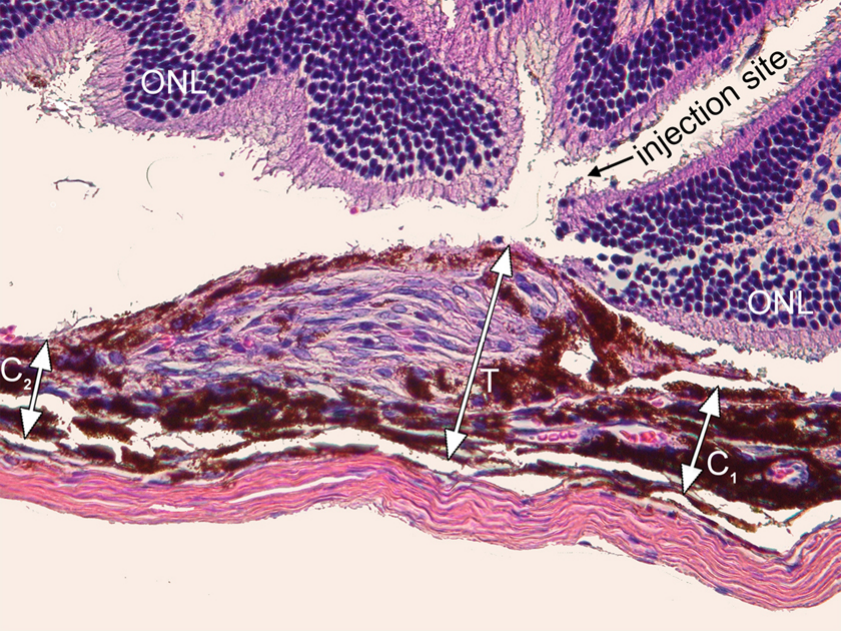
Illustration of the choroidal neovascularization thickness measurement technique. The photomicrograph demonstrates a representative choroidal neovascularization (CNV) membrane CNV 14 days after subretinal injection of retinal pigment epithelium (RPE) cells. The distance between the outer surface of the choroid and the inner surface of the CNV, defined as T, and the distance between the inner border of the choroid and the RPE monolayer next to the CNV, defined as C1 and C2, respectively, are indicated by arrows. The relative thickness of the CNV membrane, defined as R, was calculated as follows: R=(T-C)/C. C=(C1+C2)/2. Original magnification is 40X.

Freshly enucleated eyes (n=5 at each time point from PI day 3, 7, 10, 14, and 21) were fixed in 10% buffered formalin for 24 h, dehydrated in a series of graded alcohols, and embedded in paraffin. Serial sections of 6 μm thickness were cut and stained with hematoxylin and eosin according to routine protocols [37]. All tissue sections were analyzed for the presence of CNV lesions, and the individual CNV growth pattern (sub-RPE space, subretinal space, or both) was recorded. For CNV thickness measurements, tissue sections were masked and evaluated by two observers (I.S., L.B.) at 20X magnification using a bright-field microscope (BHTU; Olympus, Tokyo, Japan). The section with maximal CNV membrane extension (in regard to height) was identified and analyzed. In addition, CNV thickness measurements were performed on 4 adjacent tissue sections (2 before and 2 behind the selected section with the maximal CNV extension). The exact delineation of the CNV lesions was sometimes difficult, because of intensive pigmentation of the CNV membrane and the underlying choroid. Therefore, CNV thickness was estimated indirectly by measuring the distance between the outer border of the pigmented choroid (next to the sclera) and the inner surface (adjacent to the photoreceptor outer segments) of the CNV (T) and the thickness of the intact pigmented choroid adjacent to both sites of the CNV (C; Figure 4) previously described [38,39]. The data from each group were pooled, and the mean T and C were calculated. The relative thickness of the CNV membrane was defined as a ratio of the subtraction (T minus C) over C (mean) (R=[T-C]/C).

### Immunohistochemistry and immunofluorescent studies

Immunohistochemical and immunofluorescent studies were performed using one of the following unconjugated primary antibodies: 1:2,560 anti-CD68 (clone: KP1; Dako Corp., Carpinteria, CA), 1:200 anti-cytokeratin 18 (CK 18, monoclonal, MAB3234; Chemicon International, Inc., Temecula, CA), 1:1,000 anti-von Willebrand factor (vWF; polyclonal rabbit anti-mouse, AB7356; Chemicon International), 1:200 anti-vascular endothelial growth factor (VEGF, polyclonal goat anti-mouse, sc-1836; Santa Cruz Biotechnology, Inc., Santa Cruz, CA), 1:200 monocyte chemoattractant protein-1 (MCP-1, polyclonal goat anti-mouse, sc-1785; Santa Cruz), 1:200 matrix metalloproteinases-2 and −9 (MMP-2, polyclonal goat anti-mouse, 1:200, sc-8835; MMP-9, polyclonal goat anti-mouse, sc-6840; Santa Cruz). Briefly, sections were deparaffinized with xylene and rehydrated in graded ethanol solutions. Antigen retrieval using citrate buffer (pH 6.0) was performed for 5 min at 120 °C followed by cooling for 10 min before immunostaining. For immunohistochemistry, sections were exposed to 3.0% hydrogen peroxide for 5 min and incubated with primary antibodies for 25 min. Binding of the primary antibodies (anti-vWF, anti-CD68) was assessed by incubation with 1:30 biotinylated secondary linking mouse- or rabbit-derived antibodies for 25 min, followed by exposure to streptavidin enzyme complex for 25 min, diaminobenzidine as a chromogen for 5 min, and counterstained with hematoxylin for 1 min. Incubation steps were performed at room temperature. Between each step, sections were washed with Tris-buffered saline buffer.

For immunofluorescence laser scanning microscopy, sections incubated overnight with following antibodies: anti-CK 18, anti-VEGF, anti-MCP-1, anti-MMP-2, and anti-MMP-9. The sections were washed and incubated for 1 h with fluorescein-isocyanate (FITC) and Texas red-conjugated secondary antibodies. The sections were then washed 3 times in PBS for 5 min each, mounted with anti-fade medium (Vectashield^®^; Vector Lab., Inc., Burlingame, CA), and coverslipped. Image acquisition was performed using a laser scanning confocal microscope (BioRad, Hercules, CA). CNV containing sections incubated with secondary antibodies only served as negative controls.

### Scanning and transmission electron microscopy

**Figure 5:**
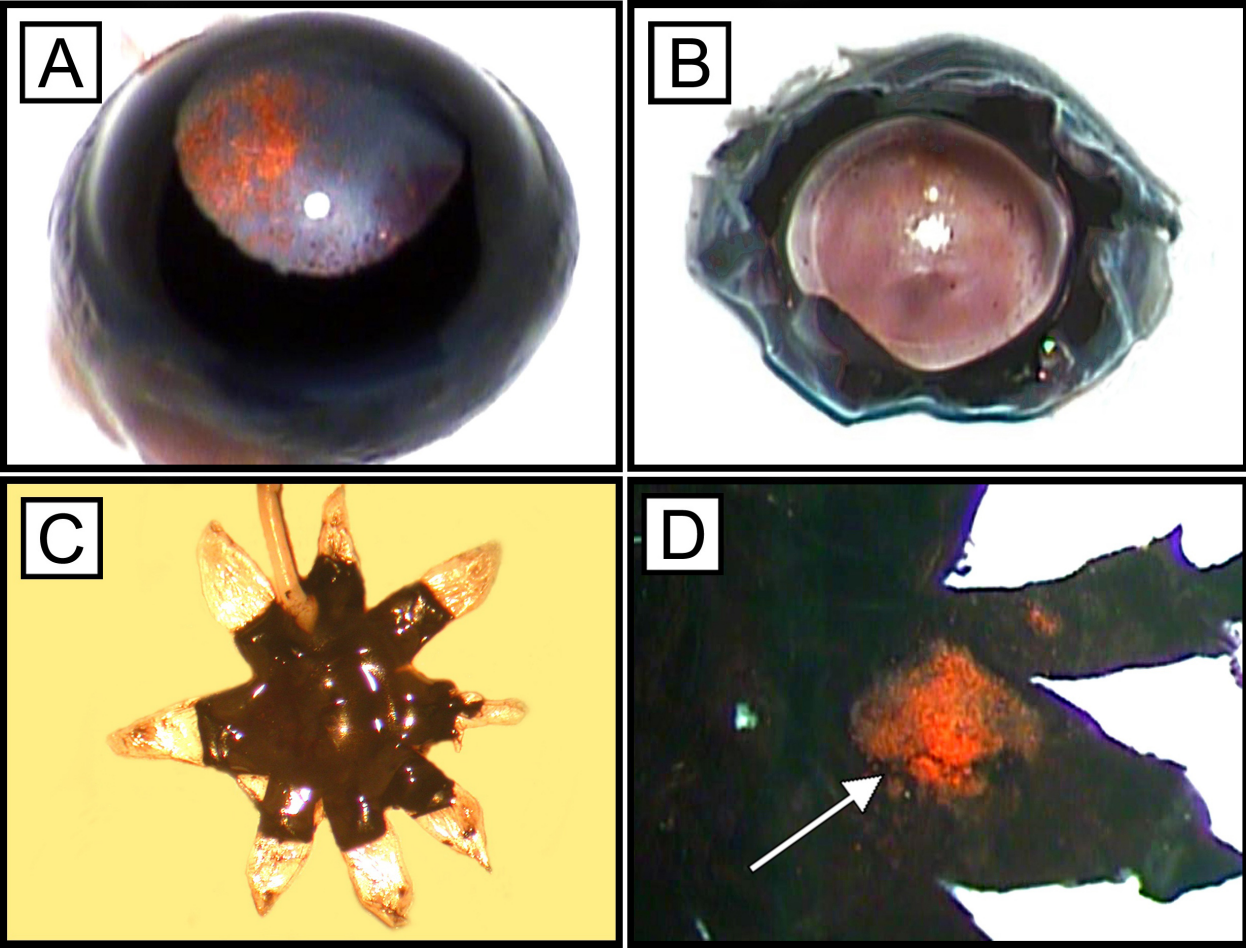
Flat-mount preparation technique. Freshly enucleated eyes (**A**) were opened by radial corneal incisions (**B**). After removal of the iris and lens, the radial relaxing incisions were extended toward the equator, and the eyes cups were flat-mounted (**C**). Choroidal neovascularization membranes (arrow) remained attached to the choroid after gentle removal of the retina (**D**)

For scanning electron microscopy, eyes were rinsed in HBSS with subsequent removal of extraocular tissues components including the optic nerve (Figure 5). The eyes were opened anteriorly by 8 radial-oriented corneal incisions, which extended from the center of the cornea to the limbus. The iris and lens were then carefully removed. The eye cup was flattened by extending radial relaxing incisions to the equator until a symmetric “starfish” appearance was achieved. Finally, the retina was dissected at the ora serrata and gently separated from the underlying RPE. The remaining eye cups containing CNV membranes, RPE, choroid, and sclera were fixed in 2.5% glutaraldehyde, rinsed in 0.1 M cacodylate buffer, and post-fixed in 1% osmium tetroxide. After dehydration, the flat-mounted eye cups with adherent CNV membranes were fixed on specific stubs, sputtered with gold-pallidium, and examined by scanning electron microscopy (JEOL 35 CF; JEOL Ltd., Tokyo, Japan) at magnifications ranging from 2,600X to 6,000X.

For transmission electron microscopy freshly enucleated eyes were fixed in 2.5% glutaraldehyde for 4 h at room temperature. Cornea, iris, and lens were removed, and the remaining eye cups, including retina, CNV, RPE, choriocapillaris, and sclera were washed with 0.1 M cacodylate buffer (pH 7.4). CNV membranes were detected using a dissecting microscope. A full thickness area, measuring 2×2 mm and centered on the CNV, was removed from the remaining eye cup. The dissected tissue block was post-fixed in 1% osmium tetroxide, dehydrated in graded ethanol, and embedded in LX-112. Serial thick sections (1.5 μm) were cut, stained with 1% toluidine blue in 1% borate buffer, and subsequently evaluated by light microscopy. The blocks were trimmed around areas of interest and thin sections (70–80 nm) were prepared. These were placed in a copper grid, double-stained with uranyl acetate and lead citrate, and examined with a JEM-100 CXII transmission electron microscope (JEOL Ltd.) at various magnifications ranging from 3,500X to 12,000X.

### Statistical analysis

Two-way ANOVA was used to compare the means of CNV thickness among study groups 1 and 2 (C57BL/6; 2-month-old mice; RPE only, RPE+microbeads) and the two control groups (C57BL/6; 2-month-old mice; PBS and microbeads only) at 5 time points (Table 2). Overall pairwise comparisons of means among the study and control groups (study groups 1 and 2, control groups 1 and 2) were made using Tukey’s multiple comparison procedure. Pairwise comparisons of means among study groups and controls at each time point were made using the Bonferroni procedure as modified by Holm [40]. The same methods were used to compare means of CNV thickness among young (2-month-old mice) C57BL/6 and ∆Ccl-2 mice (study groups 2 and 3) as well as young and aged (2-month-old mice versus 12-month-old mice) C57BL/6 mice (study groups 2 and 4), which were injected with RPE and microbeads. ANOVA was used to determine the relationship (linear, quadratic, etc.) between the CNV thickness and time in days since the start of the experiment We found that the variances of CNV thickness differed among the study groups by themselves and among the study and control groups; therefore, all analyses were done using the natural log of CNV thickness. However, means and standard deviations of CNV thickness are reported in the original units. All tests were conducted two-sided with a p value ≤0.05 considered statistically significant. The statistical calculations were done using the Statistical Analysis System (SAS, Cary, NC) software.

**Table 2:** CNV thickness measurements of the study and control groups

**Mice**			**CNV incidence** **(%/eyes)**	**CNV thickness (ratio; mean±SD)**				
**Group**	**Genotype**	**Age (month)**	**Day 3**	**Day 7**	**Day 10**	**Day 14**	**Day 21**
Study group 1	C57BL/6	2	96/24	4.9±0.6	5.8±1.8	3.3±1.0	2.7±0.5	2.3±0.8
Study group 2	C57BL/6	2	92/23	5.3±1.6	8.6±3.0	7.3±1.8	6.7±1.2	3.5±1.7
Study group 3	Ccl-2^−/−^	2	96/24	1.7±0.4	3.5±1.3	3.4±0.7	2.4±0.5	1.8±0.6
Study group 4	C57BL/6	12	96/24	2.6±0.4	3.5±0.8	3.5±0.3	2.8±1.3	1.1±0.4
Control group 1	C57BL/6	2	96/24	1.7±0.8	3.0±1.5	2.4±0.8	2.2±0.6	1.3±0.2
Control group 2	C57BL/6	2	88/22	2.0±0.5	2.3±0.5	2.3±0.1	2.5±1.0	2.5±0.5

The table summarizes the results of the choroidal neovascularization (CNV) thickness measurements of the 4 study and 2 control groups at post inoculation day 3, 7, 10, 14, and 21. Data are provided as means and standard deviations (±SD). The incidence of CNV induction was similar in all study and control groups (range 88% to 96%), however, CNV growth was superior in 2-month-old C57BL/6 mice following subretinal injections of RPE cells (study group 1) or RPE cells and microbeads (study group 2). Abbreviations: Ccl-2 knockout mice (Ccl-2^−/−^); standard deviation (SD).

## Results

### Fluorescein angiography

**Figure 6:**
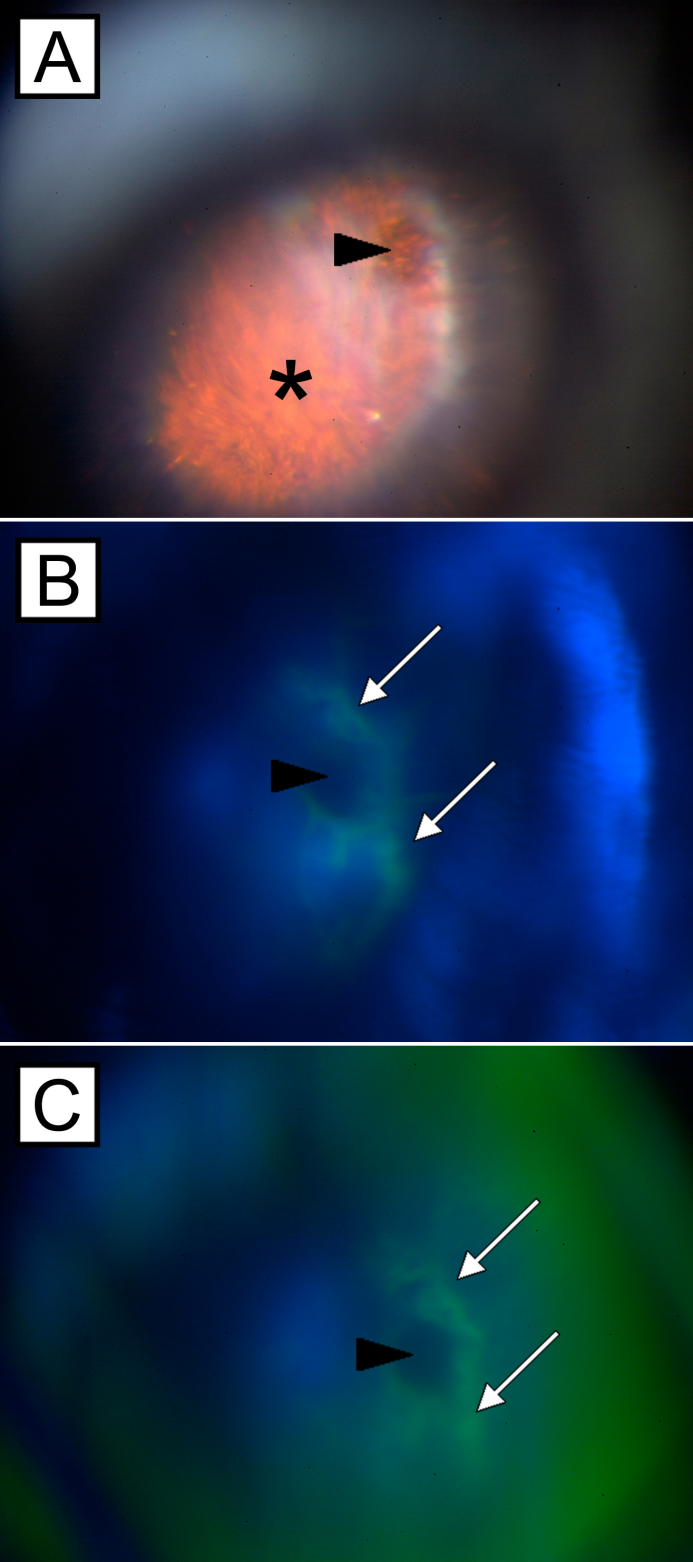
Fluorescein angiograms of a subretinal choroidal neovascularization membrane. Fundus photographs of a mouse eye with a choroidal neovascularization membrane (asterisk) 14 days after subretinal injection of microbeads. The original subretinal injection site is indicated by arrowheads (**A-C**). Sequential fluorescein angiograms (**B,C**) demonstrate fluorescein leakage (white arrows) around the injection site about 120 to 180 s after intraperitoneal installation of the fluorescein dye.

Faint fluorescein leakage occurred at the CNV membranes up to 5 min after intraperitoneal injection. Initially, fluorescein angiograms usually demonstrated a hyperfluorescent spot or ring-like configuration adjacent to the subretinal injection site. Over time, fluorescein leakage increased in size and intensity in all study groups (Figure 6). Fluorescein intensity correlated with the size of the CNV and was stronger in larger membranes. Maximum fluorescein leakage was observed around PI days 7 and 10. Older lesions (PI day 21) showed only faint fluorescein staining of the CNV with minor changes over time (data not shown).

### Light microscopic evaluation and quantitative analysis of CNV membranes

**Figure 7:**
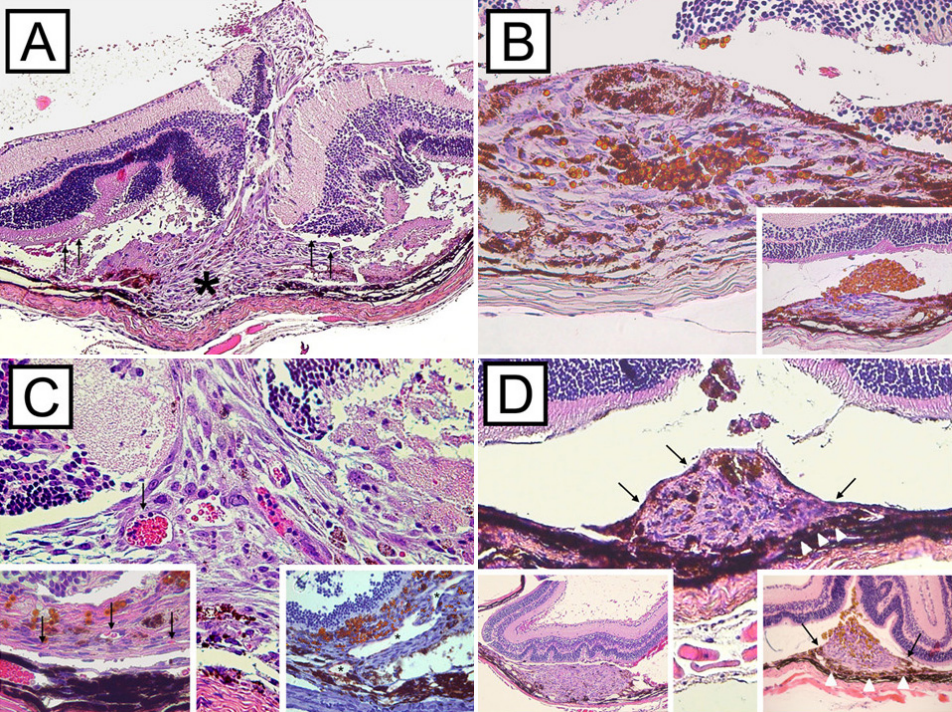
Representative hematoxylin and eosin stained cross-sections of subretinal choroidal neovascularization lesions. Membranes extended into the subretinal space between the retinal pigment epithelium (RPE) monolayer and the photoreceptor outer segments of the neurosensory retina. Focal damage of Bruch’s membrane and choriocapillaris (asterisk) were present in all lesions (**A**, image magnification 20X). Subretinal injections were often accompanied by mild inflammation and choroidal bleeding as shown in representative CNV membrane 3 days after subretinal injection of RPE cells (**A**, arrows). Microbeads and RPE cells were usually equally distributed within the CNV membranes (**B**, main image magnifaction 40X; PI day 7). In contrast, CNV membranes induced by subretinal injection of microbeads often demonstrated accumulation of microbeads at the inner surface of the lesion (**B**, insert magnification 20X; PI day 7). Newly formed blood vessels, which stained positive for von Willenbrand factor (**C**, insert bottom right, asterisks, magnification 40X; PI day 10), arose from the choroid and were present in CNV lesions from PI day 3 thru 21 (**C**, main image and insert bottom left, arrows, magnification 40X; PI day 3 and PI day 21 respectively). Initially, CNV surface was free of pigmented RPE cells (**D**, insert bottom left, PI day 3, magnification 10X). Over time (PI day 7 to 10) spindle-shaped RPE cells (arrows) started growing over the CNV membranes (**D**, insert bottom right, PI day 10, magnification 20X) and covered the lesions almost completely by PI day 21 (**D**, main image, PI day 21, magnification 20X).

CNV lesions were detected by light microscopy in the majority of the injected eyes of the 4 study and 2 control groups (Table 2). CNV membranes were composed of pigmented and non-pigmented spindle-shaped RPE cells, fibroblasts, and vascular endothelial cells. Mild accumulation of erythrocytes and inflammatory cells site were often seen in the early stages after subretinal injection within the subretinal space around the previous injection (PI day 3; Figure 7A). All lesions occurred in the subretinal space with extension between the RPE and the photoreceptor outer segments. A central break in Bruch’s membrane was seen in the majority of the membranes (Figure 7A).The retina adjacent to the inner surface of the CNV lesion displayed degenerative changes with partial loss of photoreceptor outer segments. A uniform distribution of microbeads within the CNV was observed after combined injection of RPE cells and microbeads (Figure 7B; main image); microbeads often were incorporated by adjacent RPE cells. In contrast, eyes that underwent subretinal injections of microbeads only often demonstrated separation and accumulation of microbeads at the inner surface of the CNV lesions (Figure 7B; insert). Vascularization of CNV membranes could be observed as early as PI day 3 and was present up to PI day 21 (Figure 7C). Examination of serial sections revealed that the newly formed blood vessels arose from the choriocapillaris with subsequent extension into the CNV lesions through the break in Bruch’s membrane (Figure 7C; main image, inserts). During CNV evolution, membranes were partially or completely covered by pigmented RPE cells. These cells appeared to migrate from the lateral margin of the CNV toward the center of the lesions (Figure 7D). Histopathologic findings were similar in all study groups.

**Figure 8:**
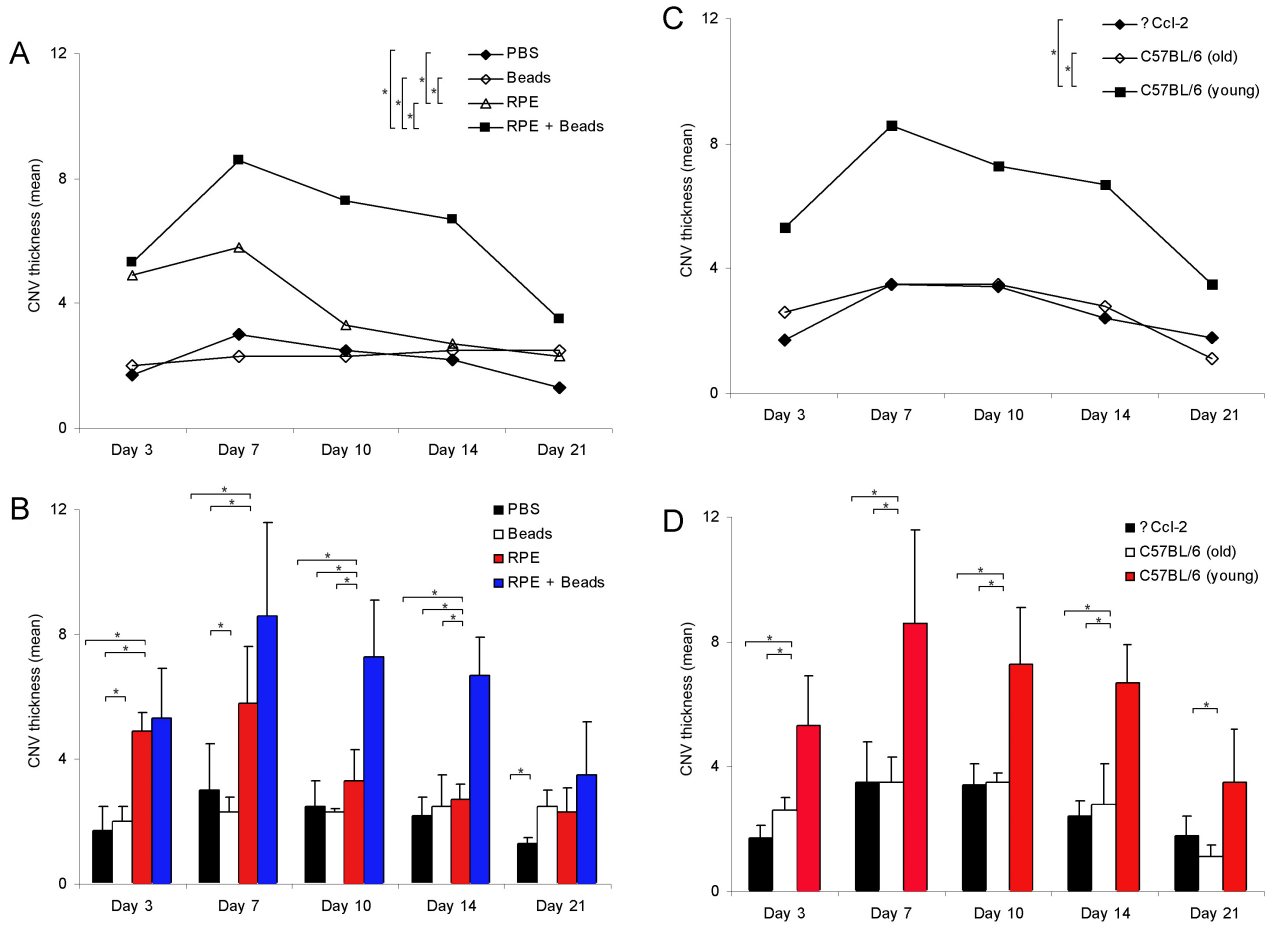
Growth dynamics of choroidal neovascularization membranes following subretinal injection. Line and bar graphs (**A-D**) display changes in the thickness of choroidal neovascularization (CNV) membranes at post inoculation (PI) day 3, 7, 10, 14, and 21. CNV formation was more pronounced in eyes following retinal pigment epithelium (RPE) cells and microbeads with maximal extension at PI day 7 (**A,B**). CNV lesions were thicker in 2-month-old C57BL/6 mice compared to age-matched Ccl-2-deficient and aged 12-month-old C57BL/6 mice **(C,D)**. Bars represent the means (n=5 eyes/group) at each time point (PI day 3, 7, 10, 14, and 21); error bars represent standard deviation (SD). The asterisk indicates p<0.05.

CNV thickness (relative) analysis was performed on serial sections through the entire CNV lesion. The measurements are based on the maximal CNV height and the results are summarized in Figure 8A-D and Table 2. Quantitative thickness measurements demonstrated dynamic changes in CNV thickness (R) during CNV evolution, which were characterized by initiation and involutional stages. Maximum thickness was observed between PI days 7 and 10 followed by subsequent regression toward PI day 21. For 2-month-old C57BL/6 mice, the two-way ANOVA showed that both subretinal injection of RPE cells as well as polystyrene microbeads (p<0.0001) and day (p=0.0002) were significant factors; the interaction of these factors was not significant (p=0.13). Mean CNV thickness was significantly different among the controls and study groups 1 and 2 (Figure 8A). Maximum CNV thickness was observed in eyes (C57BL/6) that underwent subretinal injections of RPE cells and microbeads or RPE cells alone (Figure 8A,B). In contrast, C57BL/6 mice developed only small lesions following subretinal injection of microbeads or PBS. The ANOVA showed that the changes in CNV thickness over time followed a nonlinear pattern (p=0.0017 for the quadratic term for day).

Among strains of mice treated with RPE and microbeads, the two-way ANOVA demonstrated that both strain (p<0.0001) and day (p<0.0001) were significant factors and that their interaction was not significant (p=0.76). The mean CNV thickness was significantly different over time between 2-month-old C57BL/6 (young), 2-month-old ∆Ccl-2 mice, and 12-month-old C57BL/6 mice (Figure 8C). CNV membranes in ∆Ccl-2- mice, which are characterized by impaired recruitment of monocytes and macrophages [33], showed a dynamic growth pattern similar to age-matched wild-type mice (C57BL/6; Figure 8C,D). However, the central CNV thickness was statistically smaller compared to wild-type mice at each PI day, except day 21 (Figure 8D, Table 2). No differences were observed with regard to morphologic features between both mouse strains; quantitative analysis of inflammatory cells within the CNV lesions (i.e., macrophages) was not performed. Comparison of CNV thickness measurements between young and aged C57BL/6 mice demonstrated a reduced CNV size in older animals, which were statistically significant at PI days 7 and 14 (Figure 8D, Table 2). Older animals revealed maximum CNV thickness at PI day 7 and minimal CNV central thickness at PI day 21. The ANCOVA found that changes in CNV thickness over time followed a nonlinear pattern (p<0.0001 for the quadratic term for day).

### Immunohistochemistry and immunofluorescent studies

**Figure 9:**
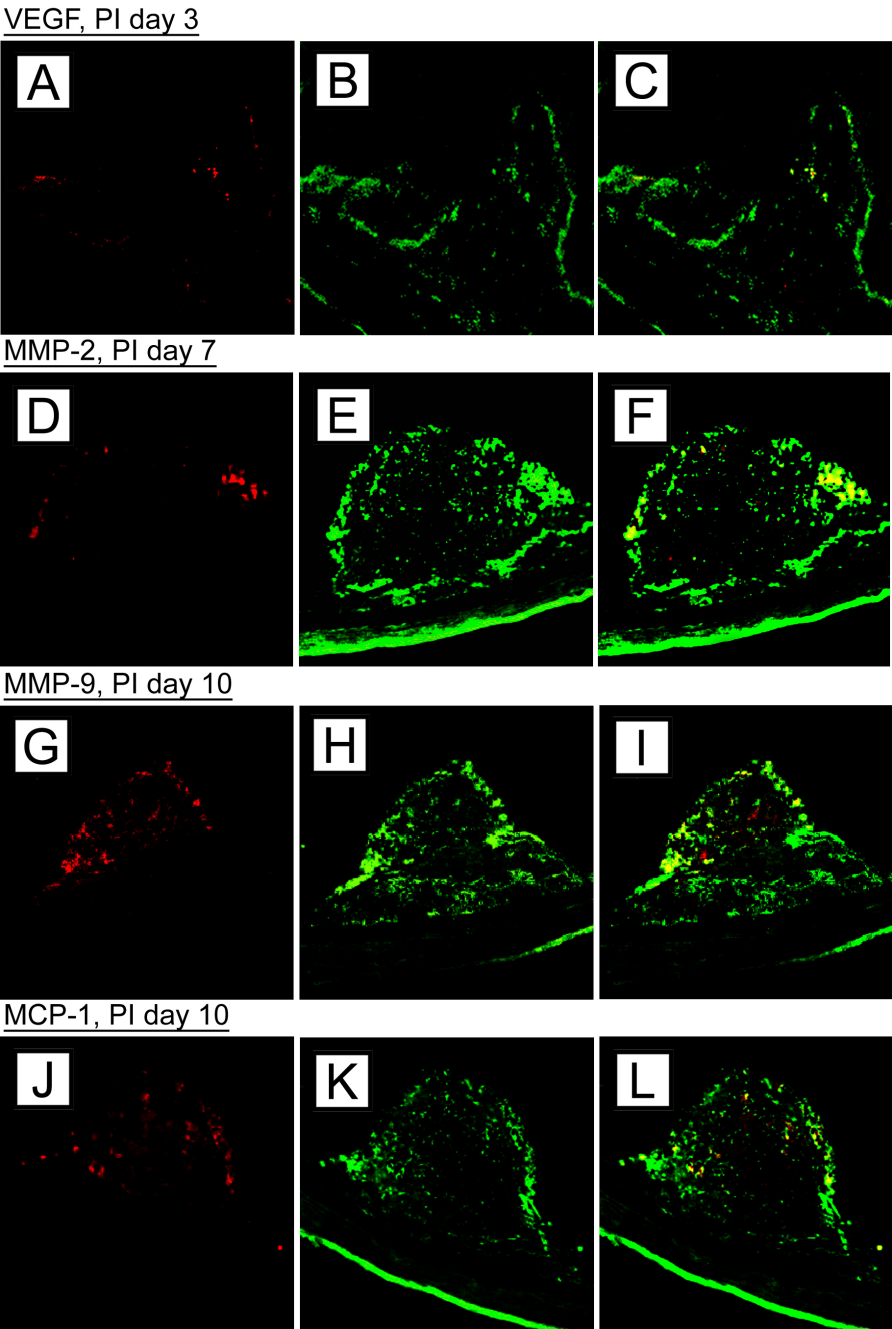
Confocal laser scanning microscopy on choroidal neovascularization membranes. Images display choroidal neovascularization (CNV) membranes of 2-month old C57BL/6 mice after subretinal injection of retinal pigment epithelium (RPE) cells and microbeads. Tissues section with CNV lesions were stained with antibodies against various cytokines and cytokeratin (CK) 18 (left column) secondary Abs only; middle is with antibody to cytokine only; right shows merged image. Cytokine expression revealed a time-dependent release of vascular endothelial growth factor (VEGF; **A-C**: post inoculation [PI] day 3), matrix metalloproteinases (MMP)-2 (**D-F**: PI day 7), MMP-9 (**G-I**; PI day 10), and monocyte chemoattractant protein (MCP)-1 (**J-L**; PI day 14) by CK 18-positive RPE cells.

Antibodies against CK 18, an intermediate filament specific for the cytoskeleton of normal and reactive RPE cells, were used to identify and localize RPE cells within the CNV lesions [36,38,39]. Dual-labeling for CK 18 and one of the following cytokines such as VEGF, MMP-2, MMP-9, and MCP-1, demonstrated immunoreactivity of RPE cells within and adjacent to CNV lesions in all study groups (Figure 9). RPE-related cytokines expression was strongest at PI day 3 and 7 and subsequently decreased. Minor cytokine secretion was observed after PI day 10.

Endothelial cells and macrophages could be detected by anti-vWF and CD68 antibodies, respectively. Quantitative analysis of cytokine expression and macrophage distribution was not performed.

### Scanning and transmission electron microscopic evaluation

**Figure 10:**
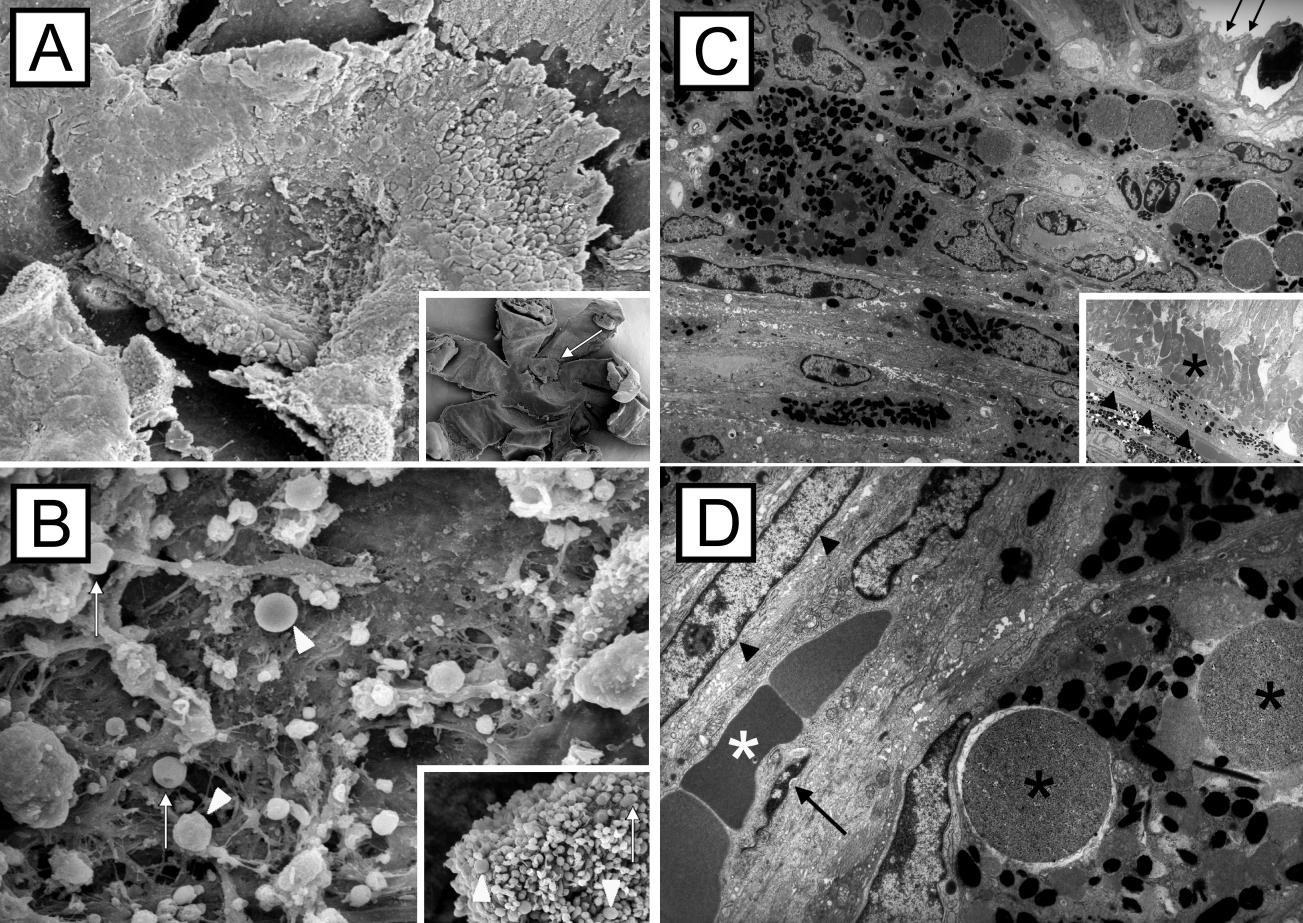
Scanning and transmission electron microscopic images of choroidal neovascularization membranes. Choroidal neovascularization (CNV) membranes (**A**, arrow) were partially covered by remaining photoreceptor cells after removal of the retina. Occasional microbeads (**B**, arrowheads) and doughnut-shaped erythrocytes are present at the inner CNV surface (**B**, arrows). CNV membranes were composed of fibroblasts, pigmented retinal pigment epithelial (RPE) cells, and non-pigmented RPE cells; a well structured surface was missing (**C**, arrows). The RPE monolayer (**C**, arrowheads) and photoreceptor outer segments (asterisks) next to the CNV margin appeared normal. High magnification images of the CNV lesions revealed pigment-laden RPE cells with intracytoplasmatic microbeads (black asterisks), spindle-shaped fibroblast (arrowheads), endothelial cells (arrow), and erythrocytes (white asterisk) within small-sized blood vessels (**D**).

Scanning electron microscopy of flat-mounted specimens revealed CNV membranes that could be easily identified and distinguished from the optic nerve head.  The lesions were elevated, with well defined margins, and their shapes were round to oval (Figure 10A). The CNV surface often revealed remnants of the adjacent neurosensory retina. High magnification images showed various round- to spindle-shaped nuclei of different sizes and photoreceptor outer segments intermixed with microbeads and erythrocytes (Figure 10B).

Transmission electron microscopy displayed a variety of cells within the CNV membranes. The lesions were mainly composed of pigment-laden RPE cells, spindle-shaped fibroblasts, and vascular endothelial cells. The endothelial cells were usually flat demonstrating occasional fenestrations. Microbeads were located either extracellular or intracellular in RPE cells (Figure 10C,D).

## Discussion

CNV development recapitulates a stereotypic wound healing response secondary to degenerative, inflammatory, and traumatic alterations of the RPE–Bruch’s membrane–choriocapillaris complex [5,21,38,39]. It comprises dynamic changes, including initiation (cytokine-driven influx of neutrophils and macrophages), stabilization (angiogenesis and extracellular matrix formation), and involution/regression stages (tissue remodeling and scarring), resulting in granulation tissue formation [14,21,41]. Current experimental CNV animal models are primarily based on laser-induced injuries (e.g., argon, krypton, or diode) to Bruch’s membrane and the choriocapillaris as well as transgenic and surgical approaches [22,25,26,29,30,38,42–45]. Although well established, present experimental CNV animal models do have potential limitations. Laser-induced CNV lesions, for example, are usually small in size and often show a mixed growth pattern (Type I and II), demonstrating retinal gliosis and retinal neovascularization [12,14,30]. In addition, the reported incidence of CNV membranes is inconsistent among different approaches [26,42].

The purpose of our study was to develop a reliable and reproducible CNV animal model easily monitored by fluorescein angiography and histopathology examination. The described CNV model is based on subretinal injections of RPE cells and chemically inert microbeads. However, the high incidence of CNV lesions even in the control groups (subretinal injections of PBS and microbeads only) indicates that a mechanical trauma to Bruch’s membrane secondary to subretinal injections seems to be the predominant factor for the CNV lesions to develop. Our findings are in accordance with previous observations that propose a compromised structural barrier between retina and choriocapillaris, even in absence of a mechanical trauma (i.e., secondary to chronic inflammation with upregulation of metalloproteinases) as mandatory for CNV formation [22,28,34,42,46–49]. Overall, subretinal injections of RPE cells, alone or in combination with microbeads, proved to be efficient and highly reliable for subretinal CNV formation with an overall incidence ranged between 88%–96%, comparable to previous investigations (54–100%) [30,31,42,50]. CNV lesions were characterized by fluorescein leakage and dynamic changes including initiation as well as active inflammatory and regression stages [10,21,38].

RPE cells were used since they represent the most common cellular component of CNV lesions and have been shown to play a pivotal role in intraocular inflammation and ocular wound healing such as CNV formation [9,15,16,18,38,51–53]. Once stimulated, RPE cells transdifferentiate into a wound-healing phenotype, releasing pro-inflammatory and angiogenic cytokines (i.e., VEGF, MCP-1, MMP-2, and MMP-9) in a time-dependent fashion as demonstrated in our model and previous models [21,33,51,52,54]. In addition, RPE cells have been shown to synthesize different proteins and receptors of the complement system that are involved in CNV formation [16–18]. Microbeads on the other hand are biologically inert, nondegradable vehicles designed for immunomagnetic cell separation and protein purification. Like microspheres and sepharose beads, they can be labeled with antibodies and small molecules (i.e., growth factors, cytokines) with subsequent delivery to different ocular compartments [23,31,55].

In comparison, combined injections of RPE cells and microbeads were most efficient in inducing CNV lesions. Differences in CNV extension of eyes injected with RPE cells only and those which received RPE cells and microbeads might in part be explained by prolonged and increased cytokine expression secondary to microbead-induced RPE cell separation [56,57]. RPE cell culture models have shown that regenerative mechanisms, such as proliferation and transdifferentiation, of RPE cells are facilitated by close cell-cell interactions and inversely correlated to cell contact inhibition [56,58]. Morphologic and ultrastructural evaluation of the induced CNV lesions showed no differences among the individual groups. CNV membranes were mainly composed of RPE cells, fibroblasts, and endothelial lined blood vessels and displayed increased fibrosis and reduced cellularity with aging [2,14]. The newly formed blood vessels originated from the choriocapillaris, although a contribution of the retinal vasculature as described in transgenic and laser-induced CNV models could not be excluded [27,28].

To further confirm our model, we also examined the effect of impaired macrophage trafficking (∆Ccl-2 versus C57BL/6 mice) and aging (12-month-old versus 2-month old C57BL/6 mice) in CNV evolution as previously performed [49,59–62]. Similar to RPE cells, macrophages are common CNV cell types and are able to promote CNV formation by expressing chemotactic, angiogenic, and proteolytic cytokines (i.e., tumor necrosis factor [TNF]-α, TGF-β, and VEGF) [5,9,12,38,61,63,64]. Macrophage recruitment to the site of inflammation is triggered by different chemotactic factors, such as MCP-1 (Ccl-2), a member of the C-C chemokine super family, which is secreted by RPE cells, astrocytes, fibroblasts, and endothelial cells [9,65–68]. A close interaction of MCP-1 and macrophages in CNV formation could be confirmed in our model that showed significantly smaller CNV lesions in Ccl-2 deficient mice compared to age-matched C57BL/6 mice. Similar results have been previously reported after systemic macrophage depletion [9,38,60,68,69]. That CNV lesions were still able to develop in MCP-1 knockout mice might be explained by co-expression of other monocytes attractant chemokines such as macrophage inflammatory protein-1α and −1β and RANTES (regulated on activation, normal T expressed and secreted) [41,62,70,71]. Resident macrophages and inflammatory cells such as neutrophils, lymphocytes, and natural killer cells, also demonstrate angiogenic capacity and thereby partially contribute to CNV formation [61,65,68,72].

Compared to Ccl-2-deficient mice, we also found smaller lesions in 12-month-old wild-type (C57BL/6) mice. In contrast to 2-month-old C57BL/6 mice, lesions were significantly smaller in thickness, revealing impaired angiogenesis and neovascularization in older animals [73,74]. Our data might in part be explained by an age-related delayed and impaired wound healing response with a reduced expression of chemokines and angiogenic factors [59,60,73–75]. Aging animals are usually characterized by a deteriorated immune system exemplified by an imbalance of pro-and anti-inflammatory mediators and detrimental effects on specific cellular components of the innate immune response [76]. In addition, aged macrophages and endothelial cells are functionally compromised, resulting in an impaired phagocytotic and proliferative capacity [75,77,78].

In summary, our experimental animal model represents a reliable and reproducible approach to initiate CNV by subretinal injection of RPE cells in combination with microbeads. CNV formation can easily be visualized by fluorescein angiography, light microscopy, and electron microscopy. Our model is applicable to various murine strains and might be used to study the effect of novel anti-angiogenic treatments by labeling those agents with microbeads. Although microsurgical skills and some initial training are required to obtain consistent results, our model appears to be less complicated in comparison to previous models as it eliminates the need for vitreoretinal surgery including vitrectomy [22,30,34]. The success rate of producing CNV is comparable to similar experimental CNV animal models [30,31,42,44,47,79,80]. Limitations of our current study include the small sample size and the short follow-up period. In addition, a direct correlation to CNV formation in humans is not possible since our mouse model is a trauma model. Modifications (i.e., labeling of microbeads with pro-angiogenic factors) as demonstrated in other studies might be of interest to further improve our model and to develop longer-lasting CNV lesions [23,31,44].
